# The effect of 4 weeks fixed and mixed intermittent hypoxic training (IHT) on respiratory metabolic and acid-base response of capillary blood during submaximal bicycle exercise in male elite taekwondo players

**DOI:** 10.20463/jenb.2016.0035

**Published:** 2016-12-31

**Authors:** Hun-young Park, Sub Sunoo, Sang-seok Nam

**Affiliations:** 1 Performance Activity and Performance Institute, Konkuk University, Seoul Republic of Korea; 2 Department of Sports Medicine, Kyung Hee University, Yongin-si Republic of Korea

**Keywords:** Male elite Taekwondo players, fixed IHT, Mixed IHT, Respiratory metabolic response, Acid-base response, Capillary blood, 80% HRmax submaximal bicycle exercise

## Abstract

**[Purpose]:**

The purpose of our study was to determine the effectiveness of 4 weeks fixed and mixed intermittent hypoxic training (IHT) and its difference from exercise training at sea-level on exercise load, respiratory metabolic and acid-base response of capillary blood during 80% maximal heart rate (HRmax) bicycle exercise in male elite Taekwondo players.

**[Methods]:**

Male elite Taekwondo players (n = 25 out of 33) were randomly assigned to training at sea-level (n = 8, control group), training at 16.5%O2 (2000 m) simulated hypoxic condition (n = 9, fixed IHT group), and training at 14.5%O2 (3000 m) up to 2 weeks and 16.5%O2 (2000 m) simulated hypoxic condition (n = 8, mixed IHT group) for 3 weeks. We compared their average exercise load, respiratory metabolic, and acid-base response of the capillary blood during 80% HRmax submaximal bicycle exercise before and after 4 weeks training.

**[Results]:**

Fixed and mixed IHT groups showed positive improvement in respiratory metabolic and acid-base response of the capillary blood during 80% HRmax submaximal bicycle exercise after 4 weeks training. However, all dependent variables showed no significant difference between fixed IHT and mix IHT.

**[Conclusion]:**

Results suggested that mixed and fixed IHT is effective in improving respiratory metabolic and acid-base response of capillary blood in male elite Taekwondo players. Thus, IHT could be a novel and effective method for improving exercise performance through respiratory metabolic and acid-base response.

## INTRODUCTION

Since the 1968 Olympic Games were held in Mexico City at 2240 m high altitude, altitude/hypoxic training has been studied for exercise performance in elite athletes^29^. Currently, this training is commonly applied to athletes and coaches with consequent improvement in exercise performance in individual and team sports^1^^, ^^40^. Erythropoiesis and exercise performance at sea-level can be improved by 2 methods through the living and training at altitude and hypoxic environments^11^^, ^^40^. First, living high training high (LHTH) was designed as way of living and training at 1,500-4,000 m high altitude environments to enhance red blood cell (RBC) count, hemoglobin (Hb) concentration, maximal oxygen consumption (VO2max), and exercise performance at sea-level. In particular, LHTH is the norm for enhanced performance in countries with natural altitude environments, including Kenya and Ethiopia^29^. Second, living high training low (LHTL) involves simultaneous beneficial effects of altitude/hypoxic acclimation (e.g., increased RBC count and Hb concentration) and sea-level training (i.e., maintenance of training intensity). In addition, LHTL has shown efficacy for enhancing athletic performance and records, resulting in positive hematological, metabolic, and neuromuscular adaptations^2^^, ^^27^. Elite athletes use living high at 2,000-3,000 m and simultaneously training low below 1,500 m ^35^^, ^^39^. However, LHTH and LHTL methods require a relatively long time (> 12 h/day and 2 weeks) in a hypoxic dose^41^. Also, LHTH is conducted in high altitude areas such as Chamonix in France, Albuquerque in United States, and Kunming in China; and LHTL is conducted with living at very expensive hypoxic accommodations and hotels^3^^, ^^22^^, ^^29^. Also, such domestic and foreign places and facilities are inadequate^27^^, ^^29^ .

In recent years, intermittent hypoxic training (IHT), a new training method at altitude/hypoxic environment, is gaining popularity. IHT involves living at sea-level and training (< 4 h/day, 2-5 times/week, and 2-4 weeks) using relatively inexpensive altitude/hypoxic environment device such as hypoxic mask and hypoxic exercise room. IHT does not improve oxygen transporting capacity of the blood by increased erythropoiesis because of exposure to relatively short interval^1^^, ^^30^. However, IHT reportedly may enhance exercise performance by stimulating an increase in glycolysis enzyme activity, glucose transporting capacity, acid-base balance capacity, skeletal muscle mitochondrial density, capillary-to-fiber ratio, and fiber cross-sectional area via upregulation of hypoxia-inducible factor 1α (HIF-1α)^7^^, ^^19^^, ^^32^^, ^^34^^, ^^38^. Therefore, IHT has an advantage of eliciting an enhanced exercise performance by positive physiological change though additional hypoxic stimulation in athletes who cannot attend altitude/hypoxic training during the season^14^. Levine^20^ and Wilber^40^ reported that IHT is more efficient in increasing exercise performance by reducing fatigue; it should be performed after reducing the hypoxic stimulation or exercise intensity at second half of training before post-test.

Besides, on exercise at high altitude and hypoxic environments, PO_2_ of the arterial blood decreases and this leads to metabolic acidosis through increased ATP synthesis by anaerobic metabolic process, increase of hydrogen ion, and decrease of pH^10^^, ^^15^^, ^^21^. In high intensity exercise, skeletal muscle fatigue is caused due to increase of hydrogen ion by response of CO_2_ and HCO_3_^-^, and damage to actin-myosin cross-bridge cycling by anaerobic glycolysis and accumulation of blood lactate, and lowering the sensitivity of calcium ion to troponin^15^^, ^^25^^, ^^26^^, ^^36^. Thus, it is important to review the pre and post changes in respiratory metabolic and acid-base response of the blood during submaximal exercise to explain the improved exercise performance. However, previous domestic and foreign studies on the effectiveness of IHT on acid-base response of the blood during submaximal exercise are inadequate.

Therefore, the purpose of our study was to determine the effectiveness of 4 weeks fixed and mixed IHT and its difference from exercise training at sea-level on exercise load, respiratory metabolic and acid-base response of the capillary blood during 80% HRmax bicycle exercise in male elite Taekwondo players.

## METHODS

### Participants

Our study included 33 male elite Taekwondo players who did not participate in any exercise and training program at hypobaric hypoxia and normobaric hypoxia environments in the previous 6 months. The participants were non-smokers, and without history of musculoskeletal, cardiovascular, or pulmonary disease. The participants received information about the purpose, process and, possible adverse effects and provided written consent prior to the start of the study. Participants were randomly assigned to training at sea-level during 4 weeks (n=8, control group), training at 16.5%O_2_ (2000 m) simulated hypoxic condition during 4 weeks (n = 9, fixed IHT group), and training at 14.5%O_2_ (3000 m) up to 2 weeks and 16.5%O_2_ (2000 m) simulated hypoxic condition (n = 8, mixed IHT group) for 3 weeks. Twenty-five of the participant completed the study (>95% compliance) and were subjected to analyses. Data from the remaining 5 subjects were discarded due to medication, withdrawal, and noncompliance. There were no significant differences in physical characteristics among groups before training (Table 1). All procedures were in accordance with the ethical standards of the responsible committee on human experimentation and with the Helsinki Declaration.

**Table 1.** Participant characteristics

	C group	F group	M group
*N*	8	9	8
Age (years)	20.50 ± 1.07	19.33 ± .87	19.88 ± .99
Height (cm)	175.50 ± 5.53	175.11 ± 4.70	171.25 ± 6.59
Weight (kg)	68.98 ± 7.13	67.97 ± 7.50	65.71 ± 6.39
Free fat mass (kg)	57.96 ± 7.84	55.90 ± 5.75	54.35 ± 6.47
Fat mass (kg)	11.01 ± 3.31	12.07 ± 6.86	11.36 ± 5.98

### Experimental design

Twenty-five male elite Taekwondo players in control group (n = 8), fixed IHT group (n = 9), and mixed IHT group (n = 8) performed training session corresponding to 80% HRmax at each environment during 4 weeks. Training session consisted of treadmill (30 min) and bicycle (30 min) exercise at a total duration of 1 h, 3 days per week, for 4 weeks. The exercise intensity set to 80% HRmax can lead to physiological changes associated with exercise performance and improved time trial in exercise training at hypoxic condition^22^. HRmax was determined using the predicted HRmax formula (Male = 206 – 0.69 age)^24^. We designed a study to measure the effectiveness of IHT group vs. control group. Therefore, at pre and post, we analyzed average exercise load, respiratory metabolic (minute ventilation; VE, oxygen consumption; VO_2_, carbon dioxide excretion; VCO_2_, respiratory exchange rate; RER, lactate blood level) and acid-base response of the capillary blood (oxygen saturation; SCO_2_, oxygen partial pressure; PO_2_, carbon dioxide partial pressure; PCO_2_, potential of hydrogen; pH, bicarbonate ion; HCO_3_^-^) during 80% HRmax submaximal bicycle exercise (5, 10, 15, and 30 min).

A nitrogen generator (Separation & Filter Energy Technology Cooperation, Korea) was used to produce normobaric hypoxic environments. The various normobaric hypoxic conditions were simulated by introducing nitrogen into the environmental chamber (width 6.5 m × length 7.5 m × high 3 m), using a nitrogen generator with the capacity to simulate normobaric hypoxic conditions for altitudes of up to 6000 m. The temperature within the environmental chamber was maintained at 20 ± 2oC and the humidity was maintained at 60 ± 2% for all conditions.

### Measurements

#### Body composition

All participants fasted overnight prior to measurement of body composition (i.e., height, weight, free fat mass, and body fat percentage). They wore lightweight clothing and were asked to remove any metal items. An X-SCAN PLUS (Jawon medical, Korea) was used to measure height and body composition.

#### Respiratory metabolic response

Respiratory metabolic response measurements were performed at 5 min, 10 min, 20 min, and 30 min over the duration of the exercise protocol. VE, VO_2_, VCO_2_, and RER were measured with the Vmax-229 breath-by-breath auto metabolism analyzer (SensorMedics, USA), the Combi 75XLⅡ(Combi, Japan) and breathing valve in the facemask form. For the blood lactate level, 80 μL blood was collected in a capillary tube using the fingertip method, and the sample was analyzed using the YSI-1500 lactate analyzer (YSI Inc., USA).

#### Acid-base response of the capillary blood

Acid-base response measurements were performed at 5 min, 10 min, 20 min, and 30 min over the duration of the exercise protocol. SCO_2_, PO_2_, PCO_2_, pH, and HCO_3_^-^ were measured by using the Gem Premier 3000 blood gas analyzer (Radiometer, USA). For the analysis of acid-base response, we collected blood samples by finger-tip method in the capillary vessel. Acid-base response of the capillary blood is similar to the results of the arterial blood and guarantee the reliability of results^36^^, ^^42^; also, it is an easier sampling method, as compared to arterial blood^6^. We collected blood samples by finger-tip method after heating the capillary vessel to 43 o Celsius using electric pad and massage for influx of arterial blood, based on previous reports on heating the blood collection region and using stimulants such as irritant cream, glyceryl trinitrate paste, and nicotinate paste^8^^, ^^9^^, ^^18^^, ^^31^. Immediately after blood sampling, change of acid-base response was evaluated within 10 s of closing front and back of the collection tube to prevent reaction of blood sample with the gases in the atmosphere.

### Statistical analysis

All data were presented as means ± standard deviations. Two-way repeated analysis of covariance (ANCOVA) was used to determine the interaction and main effects between times and groups during exercise. Post-hoc test between times in each group, and paired t-test and post hoc test between groups at each time was for LSD (least significant difference) was conducted. All statistical analyses were performed using SPSS version 22.0 (IBM Corp., Armonk, NY) for Windows. The level of significance was set at p < .05.

## RESULTS

### Change in bicycle exercise load

Bicycle exercise load to submaximal exercise at pre and post was presented in Table 2. No significant interaction and main effect was detected, and all groups showed a similar increasing rate (control group = 11.9%, fixed IHT group = 11.4%, mixed IHT group = 11.8%) in bicycle exercise load.

**Table 2.** Changes in average load of the bike exercise in each group at pre and post

Group	Pre	Post	F-value	
C	134.54 ± 25.59	150.68 ± 29.86	Time	4.023
F	132.31 ± 26.00	147.38 ± 23.66	Group	.020
M	133.09 ± 30.19	148.81 ± 30.20	Time×Group	.020

### Change in respiratory metabolic response

Respiratory metabolic response variables measured during submaximal exercise throughout the training were shown in Table 3-7. VE presented significant main effect within time and significant increase in control group at 10 min exercise. Overall, VE showed lower tendency of increase rate in fixed and mixed IHT groups, as compared with control group (Table 3). VO_2_ presented significant main effect within time at 10, 20, and 30 min exercise. Post-hoc analyses showed significantly increased VO_2_ in all groups at 10 and 30 min exercise and significant increase in control group and fixed IHT group at 20 min exercise (Table 4). VCO_2_ presented significant interaction between condition and time at 5, 10, and 20 min exercise and significant main effect within time at 10, 20, and 30 min exercise. The results of post-hoc were as follows. At 5 min exercise, fixed IHT group showed significantly lower value at post, as compared with control group. At 10 min exercise, VCO_2_ showed significant increase in control group and significantly higher value in control group, as compared with fixed and mixed IHT group at post. At 20 min exercise, VCO_2_ showed significant increase in control group and fixed IHT group, and significantly lower value in mixed IHT group, as compared with control group and fixed IHT group at post. At 30 min exercise, there was significant increase in control group and fixed IHT group (Table 5). RER presented significant main effect within time at all exercise times. By posthoc analyses, control group showed significant increase at 30 min exercise and fixed IHT group showed significant increase at 5 min exercise (Table 6). Blood lactate level presented significant main effect within time at 5 min and 20 min exercise. The results of post-hoc analyses indicated significant increase in blood lactate level in all groups at 5 min exercise (Table 7).

**Table 3.** Changes in minute ventilation (L/min) during exercise in each group at pre and post

	Group	Pre	Post	F-value	
Exercise 5 min	C	43.97 ± 10.71	48.57 ± 9.45	Time	2.219
	F	45.11 ± 7.97	43.22 ± 13.39	Group	1.538
	M	39.80 ± 10.75	40.85 ± 7.81	Time×Group	1.538
Exercise 10 min	C	73.43 ± 12.32	95.09 ± 23.66^#^	Time	8.005*
	F	78.18 ± 18.64	87.10 ± 11.98	Group	1.494
	M	76.89 ± 28.61	86.31 ± 26.11	Time×Group	1.494
Exercise 20 min	C	64.51 ± 8.37	84.26 ± 19.47	Time	2.478
	F	67.37 ± 13.53	82.42 ± 13.51	Group	1.856
	M	68.81 ± 24.80	76.53 ± 26.32	Time×Group	1.856
Exercise 30 min	C	61.60 ± 14.34	80.78 ± 15.52	Time	3.545
	F	63.31 ± 12.75	78.11 ± 14.08	Group	0.264
	M	59.75 ± 18.70	75.62 ± 25.57	Time×Group	0.264

^*^ : significant interaction or main effect

^#^ : significant difference between pre and post

**Table 4.** Changes in minute ventilation (L/min) during exercise in each group at pre and post

	Group	Pre	Post	F-value	
Exercise 5 min	C	25.63 ± 5.00	29.13 ± 2.26	Time	1.420
	F	26.84 ± 5.14	26.27 ± 9.05	Group	1.720
	M	25.91 ± 6.43	26.32 ± 5.33	Time×Group	1.720
Exercise 10 min	C	37.16 ± 6.16	47.55 ± 6.33^#^	Time	9.404*
	F	40.26 ± 7.93	46.09 ± 8.41^#^	Group	2.144
	M	40.06 ± 9.89	44.85 ± 8.77^#^	Time×Group	2.144
Exercise 20 min	C	33.94 ± 5.39	42.53 ± 4.13^#^	Time	4.944*
	F	36.07 ± 6.02	42.55 ± 5.80^#^	Group	2.158
	M	36.83 ± 8.40	40.62 ± 10.27	Time×Group	2.158
Exercise 30 min	C	31.86 ± 5.37	42.57 ± 6.24^#^	Time	5.527*
	F	35.48 ± 5.79	41.53 ± 6.59^#^	Group	3.113
	M	36.00 ± 9.31	40.05 ± 9.59^#^	Time×Group	3.113

^*^ : significant interaction or main effect

^#^ : significant difference between pre and post

**Table 5.** Changes in carbon dioxide excretion (L/min) during exercise in each group at pre and post

	Group	Pre	Post	F-value	
Exercise 5 min	C	1.59 ± 0.39	1.81 ± 0.34	Time	2.532
	F	1.71 ± 0.34	1.54 ± 0.52^a^	Group	3.671*
	M	1.54 ± 0.45	1.58 ± 0.31	Time×Group	3.671*
Exercise 10 min	C	2.71 ± 0.65	3.44 ± 0.73^#^	Time	16.935*
	F	2.76 ± 0.63	2.99 ± 0.46^a^	Group	3.743*
	M	2.68 ± 0.82	2.93 ± 0.49^a^	Time×Group	3.743*
Exercise 20 min	C	2.25 ± 0.37	2.77 ± 0.49^#^	Time	8.199*
	F	2.31 ± 0.38	2.64 ± 0.40^#^	Group	4.520*
	M	2.26 ± 0.63	2.40 ± 0.48^a^	Time×Group	4.520*
Exercise 30 min	C	2.11 ± 0.48	2.71 ± 0.54^#^	Time	8.019*
	F	2.16 ± 0.40	2.54 ± 0.49^#^	Group	1.517
	M	2.14 ± 0.69	2.46 ± 0.60	Time×Group	1.517

^*^ : significant interaction or main effect

^#^ : significant difference between pre and post

^a^ : significant difference from C group

**Table 6.** Changes in carbon dioxide excretion (L/min) during exercise in each group at pre and post

	Group	Pre	Post	F-value	
Exercise 5 min	C	0.942 ± 0.086	0.881 ± 0.056	Time	15.455*
	F	0.910 ± 0.062	0.874 ± 0.067^#^	Group	1.179
	M	0.927 ± 0.096	0.915 ± 0.065	Time×Group	1.179
Exercise 10 min	C	1.054 ± 0.065	1.023 ± 0.055	Time	56.715*
	F	1.025 ± 0.060	1.008 ± 0.045	Group	0.499
	M	1.021 ± 0.109	1.028 ± 0.022	Time×Group	0.499
Exercise 20 min	C	0.963 ± 0.050	0.932 ± 0.041	Time	49.756*
	F	0.931 ± 0.054	0.931 ± 0.030	Group	0.139
	M	0.944 ± 0.100	0.940 ± 0.052	Time×Group	0.139
Exercise 30 min	C	0.956 ± 0.037	0.902 ± 0.035^#^	Time	66.460*
	F	0.905 ± 0.056	0.912 ± 0.025	Group	2.547
	M	0.912 ± 0.103	0.935 ± 0.044	Time×Group	2.547

^*^ : significant interaction or main effect

^#^ : significant difference between pre and post

**Table 7.** Changes in blood lactate level (mmol/L) during exercise in each group at pre and post

	Group	Pre	Post	F-value	
Exercise 5 min	C	1.78 ± 0.35	1.10 ± 0.20^#^	Time	4.745*
	F	1.63 ± 0.43	1.15 ± 0.36^#^	Group	0.308
	M	1.69 ± 0.44	1.14 ± 0.32^#^	Time×Group	0.308
Exercise 10 min	C	3.84 ± 0.72	3.61 ± 1.06	Time	3.606
	F	4.24 ± 1.36	3.55 ± 0.99	Group	0.871
	M	4.40 ± 1.71	3.48 ± 1.21	Time×Group	0.871
Exercise 20 min	C	3.21 ± 0.75	3.13 ± 0.91	Time	4.405*
	F	3.50 ± 1.21	3.18 ± 0.94	Group	0.078
	M	3.45 ± 1.61	3.11 ± 1.45	Time×Group	0.078
Exercise 30 min	C	2.30 ± 0.66	2.54 ± 1.06	Time	3.096
	F	2.56 ± 1.04	2.58 ± 0.91	Group	0.107
	M	2.37 ± 1.07	2.69 ± 1.50	Time×Group	0.107

^*^ : significant interaction or main effect

^#^ : significant difference between pre and post

### Change in acid-base response of the capillary blood

Acid-base response variables measured during submaximal exercise throughout the training were shown in Table 8-12. All acid-base response variables (SCO_2_, PO_2_, PCO_2_, pH, HCO_3_^-^) showed no significant interaction effect. SCO_2_ presented significant main effect within time at 20 and 30 min exercise. By post-hoc analyses, SCO_2_ showed significant decrease in control group at 20 and 30 min exercise (Table 8). PO_2_ presented significant main effect within time at 10, 20, and 30 min exercise. In addition, PO_2_ showed significant decrease in control group at 10 and 30 min exercise (Table 9). PCO_2_ presented significant main effect within time at 10 min exercise, without significant difference in all groups (Table 10). No significant interaction and main effect in pH was detected (Table 11). HCO_3_^-^ presented significant main effect within time at 10 min exercise. Post-hoc analyses indicated significant decrease in HCO_3_^-^ in control group at 10 min exercise (Table 12).

**Table 8.** Changes in oxygen saturation (%) of the capillary blood during exercise in each group at pre and post

	Group	Pre	Post	F-value	
Exercise 5 min	C	95.25 ± 1.04	93.78 ± 1.76	Time	0.933
	F	95.22 ± 1.39	94.78 ± 0.83	Group	0.966
	M	94.50 ± 0.93	93.73 ± 2.25	Time×Group	0.966
Exercise 10 min	C	95.13 ± 0.84	93.23 ± 1.48	Time	3.851
	F	94.56 ± 1.59	93.98 ± 1.12	Group	1.116
	M	93.62 ± 1.51	93.21 ± 2.11	Time×Group	1.116
Exercise 20 min	C	95.38 ± 1.19	93.70 ± 1.88^#^	Time	5.576*
	F	94.56 ± 1.59	94.55 ± 0.73	Group	1.494
	M	95.00 ± 0.93	93.91 ± 1.71	Time×Group	1.494
Exercise 30 min	C	96.00 ± 0.76	94.13 ± 1.36^#^	Time	14.078*
	F	94.89 ± 1.76	94.44 ± 0.88	Group	1.217
	M	94.88 ± 1.25	94.36 ± 1.07	Time×Group	1.217

^*^ : significant interaction or main effect

^#^ : significant difference between pre and post

**Table 9.** Changes in oxygen partial pressure (mmHg) of the capillary blood during exercise in each group at pre and post

	Group	Pre	Post	F-value	
Exercise 5 min	C	78.63 ± 5.48	73.46 ± 6.14	Time	3.958
	F	77.89 ± 5.71	74.11 ± 3.37	Group	0.193
	M	75.00 ± 3.63	73.52 ± 7.92	Time×Group	0.193
Exercise 10 min	C	81.75 ± 3.11	74.11 ± 3.93^#^	Time	9.138*
	F	78.33 ± 6.80	75.12 ± 5.28	Group	0.546
	M	76.00 ± 5.37	73.63 ± 5.42	Time×Group	0.546
Exercise 20 min	C	81.25 ± 5.95	75.67 ± 5.00	Time	34.861*
	F	76.11 ± 6.01	76.55 ± 3.05	Group	0.270
	M	78.38 ± 5.66	76.07 ± 3.55	Time×Group	0.270
Exercise 30 min	C	80.75 ± 4.71	73.66 ± 3.94^#^	Time	19.522*
	F	76.33 ± 5.83	73.78 ± 3.80	Group	0.960
	M	76.12 ± 4.22	74.33 ± 2.82	Time×Group	0.960

^*^ : significant interaction or main effect

^#^ : significant difference between pre and post

**Table 10.** Changes in carbon dioxide partial pressure (mmHg) of the capillary blood during exercise in each group at pre and post

	Group	Pre	Post	F-value	
Exercise 5 min	C	43.88 ± 3.60	43.55 ± 2.95	Time	2.942
	F	43.33 ± 2.06	41.00 ± 2.60	Group	2.027
	M	44.25 ± 2.25	43.18 ± 3.09	Time×Group	2.027
Exercise 10 min	C	40.38 ± 3.29	40.22 ± 3.52	Time	22.107*
	F	38.78 ± 3.11	38.00 ± 1.32	Group	3.026
	M	40.38 ± 4.98	40.87 ± 2.95	Time×Group	3.026
Exercise 20 min	C	38.88 ± 3.40	37.47 ± 4.25	Time	3.595
	F	38.33 ± 1.94	35.78 ± 1.39	Group	1.239
	M	38.00 ± 4.69	37.30 ± 3.81	Time×Group	1.239
Exercise 30 min	C	39.88 ± 2.85	37.55 ± 3.12	Time	2.373
	F	38.33 ± 2.18	36.22 ± 1.64	Group	0.360
	M	38.63 ± 3.8 5	37.21 ± 3.72	Time×Group	0.360

^*^ : significant interaction or main effect

^#^ : significant difference between pre and post

**Table 11.** Changes in potential of hydrogen of the capillary blood during exercise in each group at pre and post

	Group	Pre	Post	F-value	
Exercise 5 min	C	7.383 ± 0.023	7.388 ± 0.013	Time	2.261
	F	7.397 ± 0.026	7.401 ± 0.025	Group	1.160
	M	7.388 ± 0.022	7.380 ± 0.037	Time×Group	1.160
Exercise 10 min	C	7.336 ± 0.030	7.318 ± 0.038	Time	0.404
	F	7.347 ± 0.033	7.348 ± 0.038	Group	0.909
	M	7.347 ± 0.037	7.332 ± 0.054	Time×Group	0.909
Exercise 20 min	C	7.378 ± 0.025	7.341 ± 0.039	Time	0.335
	F	7.376 ± 0.035	7.359 ± 0.031	Group	0.912
	M	7.371 ± 0.041	7.358 ± 0.062	Time×Group	0.912
Exercise 30 min	C	7.395 ± 0.014	7.367 ± 0.032	Time	0.205
	F	7.402 ± 0.033	7.380 ± 0.032	Group	0.169
	M	7.404 ± 0.023	7.373 ± 0.052	Time×Group	0.169

**Table 12.** Changes in bicarbonate ion (mmol/L) of the capillary blood during exercise in each group at pre and post

	Group	Pre	Post	F-value	
Exercise 5 min	C	26.33 ± 1.01	25.81 ± 1.33	Time	1.721
	F	26.63 ± 1.47	25.44 ± 1.65	Group	0.300
	M	26.71 ± 1.26	25.65 ± 2.01	Time×Group	0.300
Exercise 10 min	C	21.95 ± 1.21	20.79 ± 1.84^#^	Time	8.239*
	F	21.66 ± 2.71	20.94 ± 2.13	Group	1.078
	M	21.79 ± 3.94	21.76 ± 2.96	Time×Group	1.078
Exercise 20 min	C	22.83 ± 1.46	20.83 ± 1.25	Time	2.423
	F	22.51 ± 2.29	20.22 ± 1.81	Group	0.570
	M	22.25 ± 4.16	20.87 ± 3.99	Time×Group	0.570
Exercise 30 min	C	24.41 ± 1.53	21.51 ± 1.87	Time	1.023
	F	23.91 ± 2.12	21.49 ± 1.97	Group	0.351
	M	23.81 ± 3.76	21.93 ± 4.02	Time×Group	0.351

^*^ : significant interaction or main effect

^#^ : significant difference between pre and post

## DISCUSSION

Exercise at high altitude and hypoxic environments, results in a decrease in PO_2_ of the arterial blood, which leads to metabolic acidosis through increase of ATP synthesis by anaerobic metabolic process, increase of hydrogen ion, and decrease of pH^10^^, ^^15^^, ^^21^. High intensity exercise causes skeletal muscle fatigue due to hydrogen ion increase by CO_2_ and HCO_3_^-^ response, damage to actin-myosin cross-bridge cycling by anaerobic glycolysis and accumulation of blood lactate, and lowering the sensitivity of the calcium ion to troponin^15^^, ^^25^^, ^^26^^, ^^36^. Thus, review of the changes in respiratory metabolic and acid- base response of the blood during submaximal exercise at pre and post are important to explain the improved exercise performance. However, previous research regarding acid-base response of the blood has focused on changes in one-off exercise and effect of ergogenic acids such as L-carnitine, BCAA, and sodium bicarbonate during exercise and recovery in various participants^17^^, ^^26^. Also, previous studies on high altitude and hypoxic environments investigated difference between sea-level and various hypoxic conditions during exercise without change in hypoxic training such as LLTH, LHTL, IHT, and IHE^4^^, ^^25^^, ^^37^. Therefore, we investigated the effectiveness of 4 weeks fixed and mixed IHT on respiratory metabolic and acid- base response of the blood during 80% HRmax bicycle exercise in male elite Taekwondo players.

The results of our study indicated that bicycle exercise load to submaximal exercise showed a similar tendency toward increase in all groups (control group = 11.9%, fixed IHT group = 11.4%, mixed IHT group = 11.8%), with no difference between groups at pre and post. In respiratory metabolic response, fixed and mixed IHT groups presented significantly lower VCO2 at post, lower tendency for increase in VE and VO_2_, and higher tendency for decrease in blood lactate level, as compared with control group. In acid-base response of the blood, fixed and mixed IHT groups presented lower tendency for decrease in SCO_2_, PO_2_, and HCO_3_^-^, as compared with control group. However, PCO_2_ and pH showed no difference between groups.

Generally, IHT increases glycolysis enzyme activity, glucose transporting capacity, mitochondria density, capillary density, cross section area of skeletal muscle, and activity of the motor unit by stimulating the neuromuscular system. These positive changes improve the effectiveness of oxygen utilization^12^^, ^^23^^, ^^32^^- ^^34^^, ^^38^. Also, exercise performance is enhanced by improvement of inflow rate in oxygen to skeletal muscle tissue and oxygen utilization capacity in mitochondria^13^^, ^^28^. In our study, fixed and mixed IHT groups showed positive results in VE, VO_2_, VCO_2_, blood lactate level, PO_2_, SCO_2_, and HCO_3_^-^ , as compared with control group despite same increase rate on bicycle exercise load at 80% HRmax through training in all groups. Especially, positive changes of VO_2_ and SCO_2_ during exercise of the same load in our study were consistent with results of previous studies that IHT improves exercise economy such as oxygen utilization capacity in skeletal muscle tissue^12^^, ^^16^^, ^^28^^, ^^30^. It is likely that enhanced exercise economy through IHT improves PO_2_, blood lactate level, and HCO_3_^-^ by increased aerobic energy metabolic rate and consolidated tolerance and removal capacity to fatigue-causing substance during anaerobic energy metabolism^26^^, ^^28^^, ^^30^. Also, improvement of VE and HCO_3_^-^ in mixed and fixed IHT groups was possibly caused by inhibition of sympathetic nervous system, hyperactivity of parasympathetic nervous system, and adaptation of chemical receptors (carotid bodies and aortic bodies) in the central and peripheral nerve through continuous stimulation of hypoxic ventilatory response^4^^, ^^28^.

We verified the effect of IHT on respiratory metabolic and acid-base response of the blood as well as efficiency difference between mixed IHT (training at 16.5%O_2_-2000 m simulated hypoxic condition during 4 weeks) and fixed IHT (training at 14.5%O_2_-3000 m up to 2 weeks and 16.5%O_2_-2000 m simulated hypoxic condition for 3 weeks). Our study was based on previous reports that the effect of IHT is greatly influenced by hypoxic environment condition and training intensity, and IHT should be performed after reducing hypoxic stimulation or exercise intensity at second half of training before post-test to reduce fatigue. However, no significant difference in all dependent variables occurred between fixed IHT group and mixed IHT group. These results are possibly due to mixed IHT group training at the more severe hypoxic condition (fixed IHT group = 2000 m vs mixed IHT = 3000 m) up to 2 weeks and training under the same hypoxic condition from 3 weeks to 4 weeks. Thus, it is likely that IHT group did not perform training after reduced hypoxic conditions or exercise intensity at second half of training before post-test to reduce fatigue, as compared with the control group.

## CONCLUSION

Our results suggested that mixed and fixed IHT is effective in improving respiratory metabolic and acid-base response of the capillary blood in male elite Taekwondo players. Thus, IHT can be considered a novel and effective method for improving exercise performance through cardiopulmonary and acid-base response. However, no significant difference in all dependent variables was detected between fixed IHT group and mixed IHT group.
